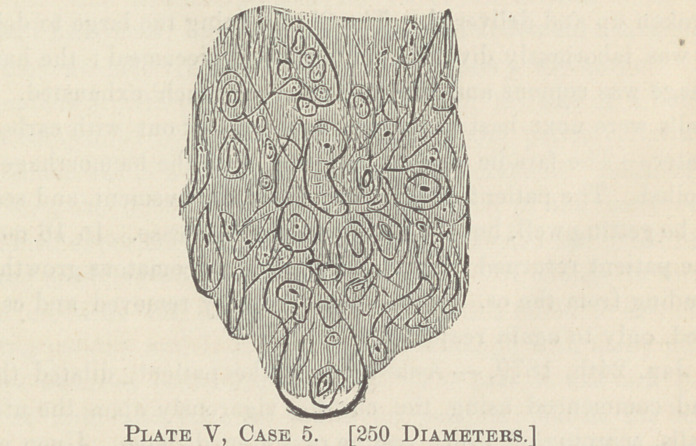# Anomalous Complications of Uterine Fibroids

**Published:** 1879-12

**Authors:** J. T. Everett


### ARTICLE IV.

### ANOMALOUS COMPLICATIONS OF UTERINE FIBROIDS. By J. T. EVERETT, A.M., M.D.

In a careful examination of the literature on uterine fibroids, there are but few references to any complications whatever. Of the few who have noticed these concomitants, sarcoma and mucous polypi seem to be most frequently mentioned by Schroeder, Nélaton and others. But our standard authors appear to ignore the connection between these troubles, or their relation, treating of each under a separate head and never referring to their synchronous appearance. Various writers have simply mentioned these complications *en passant;* but do not go into details, or dwell upon their treatment or effects. Whether these complications have proved of such small importance in their hands, or whether they are of such rare occurrence as to be seldom seen, I am unable to say. But in my hands they have entailed such sad and grave results as to be long remembered; and have occurred with such frequency as to keep the mind on the *qui vive.* Writers of our text-books may perhaps be excused from mentioning these complications *in extenso*, on the ground of economy of space and to avoid repetition, each being treated of separately under its appropriate head. But the writers of clinical cases should not omit to mention these whenever met with in their operations. The treatment of these troubles when accompanying or following fibroids is a very different matter from their treatment when occurring idiopathically. In 19 cases where I have operated for the radical removal of uterine fibroids, I have met 3 cases of mucous polypi, 4 cases of sarcomatous degeneration of the mucous membrane, and 3 where the fibroid tumor underwent retrogressive metamorphosis. Perhaps as terse and full a treatise upon this subject as in any language, is the paper by Guperon, in Billroth's "Hand-buch der Frauenkrankheiten," Stuttgart, Enke, 1878.

Schroeder, in Ziemssen's Cyclopædia of the Practice of Medicine, Vol. X. pp. 222–228, gives a very full description of the normal fibroid, and cursorily mentions, softening, induration, sarcomatisation and calcification. It is known that the process of softening and disintegration takes place by three kinds of action. 1st. By simple *œdema* thus producing atrophy of the muscular structure when normal absorption takes place. 2d. By fatty metamorphosis, where the muscular fibers undergo fatty degeneration, changing to fat globules, and these in turn being dissolved and absorbed. And 3d. By the myxomatous degeneration, where a large quantity of mucous tissue is formed among the fibers, its pressure causing atrophy of the fibers and absorption thus supervening. The indurative process is quite similar in many respects to the fatty metamorphosis. An interstitial inflammation takes place from some occult cause, and the muscular fibers degenerate into fibrinous bands, which by their contractions cause the atrophy and absorption of all softer material and the tumor assumes a hard cartilaginous appearance and ceases to grow. Of its sarcomatous degeneration, we have two modifications, one attacking the fibroid proper, the other involving the uterine tissues as well. In either case, the process seems to be the same. Round cells begin to proliferate between the bundles of muscular fibers. These continue to grow and encroach upon the surrounding tissue and gradually cause its absorption.

In my own practice of 19 operations for the removal of uterine fibroids, I have met 7 cases of sarcomatous degenerations, two of which underwent a second retrogressive transformation and finally terminated in carcinoma, and one terminated in calcification.

The conclusions of Guperon are so very pertinent to this case that I may be excused for translating the article *verbatim :*

He says: " In general it can be affirmed that the uterine fibroid is of very slow growth ; the more these consist of cellular tissue, the slower the progress and the closer their nature to true fibrous tissue.

"The muscular tumor, or the true myome, grows much faster ; it has been observed that these grow at times with astonishing rapidity ; this is peculiarly the case during pregnancy. Aside from this, the sudden voluminous swelling does not occur through rapid growth as many have stated, by enlargement, accumulation of the proper elements, but through the change in the blood accumulation, through œdema or other pathological phenomena such as inflammation. The size of the interstitial fibroid changes, especially before or after menstruation; here occurs the most striking difference, and in not a few instances has the apparent decline after the close of menstruation been mistaken for a retrogressive inflammation. Similar occurrences are readily observed in pedunculated fibroids which are extruded from the mouth of the womb, by which the pedicle is held, thereby causing swelling of the tumor through the retardation of the retroflow, producing œdema; even this must not be considered as a sudden growth. It has been observed in various exhausting and enervating diseases, that transitory diminution occurs, and at convalescence comparative rapid enlargements have occurred.

"Braun, Chaerie and Späth place their symptoms mostly during chlorotic stages. These changes are noticed by all physicians who watch their course for years. A uniform growth of the tumor is shown, yet such cases cease only to enlarge when calcification takes place."

Of the sarcomatous degeneration of uterine fibroids, the literature is not complete. The cause of the degeneration is unknown. Of its etiology and mode of occurrence there have been many theories advanced which are too visionary to repeat. The tumors seem to occur at any period in life when the preceding fibroid takes a retrogressive change. The uterine mucous membrane is sometimes first invaded by the neoplastic growth. Of the seven cases occurring in my practice, this was true in three instances. In these cases the sarcomatous proliferation appeared to spring from the mucous or sub-mucous stricture of the uterine cervix and to thicken and increase by a rapid growth of spindle-shaped cells in vast numbers, producing a soft, flabby, highly vascular tumor, which accomodates itself to the cavity it finds, extending up into the fundus and being forced out of the os by the pressure of the uterine muscles.

This growth gives rise to exhausting and almost uncontrolable hæmorrhage and various hysterical symptoms which are very troublesome to control. It may eventually end in suppuration or may penetrate the walls of the uterus and encroach upon the adjacent organs by infection and cause by absorption, grave and fatal results simulating pernicious anæmia.

PLATE I, CASE I. [250 DIAMETERS.] PLATE II, CASE 4. [250 DIAMETERS.]

The plates I and II show the microscopical structure of these neoplasms from cases I and III. The following is a history of the case:

*Case I.*—Was consulted, July 1st, 1875, by Mrs. A. S., aged 59, nullipara, who was very much debilitated from the effects of a frequent hæmorrhage, which had occurred with greater or less intensity, since the removal of a sessile fibroid, the size of an egg, two years previous. There had been a history of uterine trouble extending back for a period of 15 years. For the 16 or 18 months last passed, there had occurred between each of the hæmorrhages, a copious discharge of watery fluid containing shreds of mucous and connective tissue. Examination revealed a sessile mucous polypus, the size of a pullet's egg depending from the os. This was seized with the forceps and removed. It was composed of mucous and connective tissue filled with a mass of gelatinous blood and serum. Gave ergot, iron, quinine and strychnia.

July 18th — Discharge much less in quantity; darker and thicker in quality. The stomach rejecting the ergot it was for four days administered hypodermically. July 22d, the patient expelled eleven mucous polypi of the size and shape of small oysters. The hæmorrhage was small. Passed negative electrode into the cavity of uterus, with positive over the sacro-lumbar junction and allowed an interrupted current to flow for 15 minutes. This treatment was continued for two weeks, with benefit.

Sept. 7th.—Two more growths expelled and hæmorrhage increased; continued the faradization and the administration of iron, quinine and strychnia, and in addition the compound syrup of the hypophosphites, and ergot hypodermically.

Oct. 8th.—Seven more growths were extruded. Galvanized uterus for five minutes and faradized for twenty minutes afterwards. Same medication; patient feels better; appetite improved.

Dec. 20th.—Eleven more polypi escaped. Used the curette, but without result; no appearance of any more growths *in utero* Os rigid but walls soft and flabby.

Dec. 27th—Patient improving; hæmorrhage nearly ceased; discharge slight and clear, with no more shreds of mucous or connective tissue. Continued treatment and same medicine.

March 19th.—Patient, while out of the city on a visit, passed two or three more growths. Hæmorrhage slight; no pain.

April 1st.—Patient failing rapidly; no appetite; bowels constipated, nutrition poor; mind, despondent and gloomy. Discharge increasing and offensive; severe pain in right hip, knee and ankle.

April 11th.—No change for the better, but bad symptoms increasing in number and severity; discharge muco-purulent, containing shreds of decomposing tissue. Discontinued faradization and gave morphia hypodermically. Patient very nervous and wasted to a skeleton; medicine borne well, but nutrition entirely suspended.

June 15th.—Patient has failed very slowly; pain in right hip severe; has to be controlled by morphia; discharge copious, purulent and offensive.

July 10th.—Patient about the same, for the last month; at times seeming to rally and again falling back to below the original condition, until the 9th, when œdema appeared in the right lower limb, and is gradually creeping upwards.

July 12th.—Scarified the limb and allowed serum to escape.

Aug. 10th. — Same line of treatment, with concentrated foods and pepsine.

Sept. 2d. — Patient died to-day from exhaustion, having failed steadily since the last record. Pain continued and discharge became more free towards the last.

*Case II.* — I was consulted in the spring of 1875 by Miss R., niece of the last patient, aged 18, frail and cachectic, with lateral curvature of the spine and retroversio uteri.

These troubles were given their appropriate treatment, to which they responded slowly.

June 15th. — Upon introducing the sound to replace the retroverted uterus, a small pedunculated fibroid was found springing from the posterior portion of the fundus. The os was dilated; the growth seized with the polypus forceps, its pedicle twisted off and the body removed. The hæmorrhage was insignificant and easily controlled by the faradic current. After recovery from her complicated maladies, there still continued a slight serous discharge, and at the menstrual epoch a free hæmorrhage. This was finally corrected by a free use of faradization, ergot and iron.

Sept. 1st. — Patient passed three mucous-polypi, the size of a pullet's egg.

Threw a mixture composed of 10 C. C. tr. ferri chloridi and 10 C. C. fl. ex. a. ergota into cavity of womb. This was followed by severe pelvic pain, shock and chill, with some symptoms of peritonitis. These were easily controlled, however, by a free use of quinia and morphia. I have kept the case in view since that time, and up to the present there has been no return.

*Case III.* — I saw, in April, 1876, Mrs. B., aged 46; monipara, robust when in health, but now somewhat exsanguine from the repeated hæmorrhages, which recurred every 21 days. Severe pain in left ovary, small of the back, across the abdomen and severe gastralgia. Examination revealed sessile uterine fibroid with pedicle attached near left cornu; being closely embedded in a mass of sarcomatous tissue which sprang from the uterine mucous membrane. In the attempt to remove this growth, masses of the sarcoma were detached and forced out. The fibroid was at length secured and drawn down, its pedicle severed and the tumor removed, it being the size of a goose-egg. The curette was next used and the entire spongy mass removed, deep down into the healthy tissue ; a suppository of bromine, with simple cerate, was then introduced into the cavity of the uterus and allowed to dissolve and trickle out. This treatment produced severe burning pain, vomiting and chill; but reaction soon set in and the patient made a good recovery. In 1877, another redundant mass was removed and the uterine surface cauterized deeply. The patient has done extremely well since, and is at present apparently enjoying perfect health, with the exception of some back-ache.

Sarcomatous degeneration of the uterine fibroid proper is not of rare occurrence, as in my practice this process has followed in four cases, and in two was further complicated by a secondary change to carcinoma. Fibro-sarcoma more often attacks the body of the uterus when not affecting the tissues of a polypoid fibroid,

PLATE III, CASE 3. [250 DIAMETERS.]

and is then composed of the altered and softened muscular fibrillæ, of oval or spindle nucleated cells, amid fragments of normal or partly altered muscular fibers. Interspersed in the tissue of the neoplasm are fat globules, etc. (These conditions are imperfectly shown, by my faulty drawings, from a power of 250 diameters. Plates III, IV and V are taken from cases IV, V and VI).

*Case IV.*—I saw Mrs. B., multipara, aged 46, Sept. 15th, 1876, suffering with severe pain in the back, abdomen and head; hæmorrhage alarming and vomiting persistent. She had, from the date of the removal of a submucous fibroid three years previous, suffered for a long time with similar symptoms and a profuse watery discharge. The monthly flow had recurred with increasing frequency and severity until it was truly alarming. As the stomach would retain nothing, I at once gave hypodermic injection of ergotine, which somewhat checked the flow, but it soon

PLATE IV, CASE 6. [250 DIAMETERS.]

recurred; faradisation held the hæmorrhage in check for some time, but it recurred. Injections of ice-water shared the same fate. In desperation I threw 20 C. C. of fl. ext. ergot and tr. ferri chloridi into the uterine cavity. This at once checked the hæmorrhage and permanently; but it produced intense pelvic

PLATE V, CASE 5. [250 DIAMETERS.]

pain, chill and vomiting, with strong symptoms of peritonitis, which responded easily to central galvanism. The next day there appeared an offensive discharge from the os, and the uterus was faradized, when an immense clot was expelled, followed by some hæmorrhage, which was easily controlled by the faradic current. As soon as the stomach would retain medicine, I prescribed ergot, iron and quinine. At the next menstrual epoch, free hæmorrhage occurred, but not as much gastric trouble. This recurred two or three times, when I determined to thoroughly examine the internal surface of the womb. This revealed soft pulpy walls, and each movement of the sound produced free hæmorrhage. The os was now dilated, and with the curette its inner surface was freely scraped, and a large mass of mucous polypi removed. Two weeks afterward, a slight flow appeared, the curette was again used, but only a small amount of fungous tissue was obtained. The faradic current was used daily for some months, and ferruginous tonics with nutritious foods. The patient slowly improved, but is still troubled with pelvic tenderness, some metritic trouble and gastric irritability.

*Case V.* — February, 1877, I removed a large fibroid from Mrs. D. B., multipara, 59 years of age, robust when in health, but now exsanguine. Upon dilating the os and attempting to seize the tumor with obstetric forceps (it being so hard and large that the vulsella was useless), large masses of a sarcomatous growth were so closely packed around the tumor that the effort failed. The os was then incised and the hand introduced and the sarcoma broken up and delivered. The fibroid being too large to deliver, it was laboriously divided and removed piecemeal; the hæmorrhage was copious and the patient very much exhausted. The walls were next hastily scraped and rinsed out with carbolated water. The faradic current was used and the hæmorrhage controlled. The patient made a very rapid impovement, and seemed to be getting well, but the discharge did not cease. In 16 months the patient returned with a well marked sarcomatous growth protruding from the os. This was repeatedly removed and cauterized, only to again reappear.

Jan. 24th, 1879. — Anæsthetized the patient, dilated the os and commenced using the curette vigorously upon the uterine walls, scraping off every vestige of softened tissue. Upon working around to the right side, to my surprise, I discovered an intramural fibroid. The serrated scoop was used, and the growth enucleated, which was nearly the size of a goose-egg. The scraping was continued, and upon its completion the uterus was washed out with a strong solution of ferric chloride. The shock and chill which followed were but slight and the patient reacted well. The uterus contracted well, and soon admitted the sound, only five centimeters. The tumor was of clear white, fibrous, with some muscular and connective tissue. The neoplasm was clearly sarcomatous ; specimens of which, in connection with that of the next case were sent to Prof. Byford, of Chicago, who upon microscopical examination, confirmed the diagnosis. For some time the patient seemed to be improving and for several months the hope was indulged that the trouble was controlled. After a time, however, the fungus re-appeared accompanied by marked radiating pains and a cachectic countenance. I took the patient to Chicago, April 7th, to consult with Prof. Byford, who pronounced the present growth clearly encephaloid in character. Upon returning home, I removed every vestige of the fungus degeneration, and put the patient upon free use of arsenic. She is now apparently improving.*

*Case VI.* — Oct. 12th, 1877, I saw Mrs. H. in consultation with Dr. J. P. Anthony, aged 39, blonde and of full habit, but now exsanguine from repeated floodings. She had never been enceinte. She had complained for 16 or 18 months of pelvic pain and heaviness ; severe pain in back and abdomen, neuralgia, inter-current hæmorrhages and profuse watery discharge. A sarcomatous growth protruded from the os of the size of a hen's egg, accompanied by considerable vaginitis and pelvic cellulitis. The patient was of a peculiarly nervous temperament. Although my friend, the doctor, was an old practitioner of the most unquestioned experience and skill, the patient fancied she could not take the medicine which he had prescribed. A change in form of the medicine was therefore made ; but this failed to suit our exacting patient, and I, at her request, although with many misgivings, assumed control of the case. The patient was now put upon as mild and tasteless tonics as possible ; but at the best little medicine was taken. The faradic current was used daily with the effect of entirely checking the hæmorrhage and discharge.

* The fungus again appeared, and patient died at end of third month.

Nov. 10th.—Patient being somewhat stronger, was anæsthetized and the sarcoma removed carefully and deeply, down into the tissue of the posterior lip of the os. She improved after the operation until the second week in December, when the growth again made its appearance from above.

Dec. 18th.—She was again anæsthetized and the os dilated when, upon removing a portion of the redundant growth, a fibropolypus, the size of a goose-egg was discovered. This was seized and twisted off and all the neoplastic growth removed with the curette. Patient rallied slowly from this operation; but after a time, her appetite improved, the movements became more regular and the countenance assumed a more florid hue, showing that the red globules were increasing in number.

Jan. 4th.—The sarcoma again developed to the size of half a hen's egg. This was again removed, but not as thoroughly as was desirable as the patient refused to inhale an anæsthetic, and a hypodermic injection of morphia was given in its place. The attempt was next made to destroy the growth by applying daily, to the seat of trouble, a solution of bromine in alcohol 1 to 5. This seemed only to retard the growth and not to destroy it. From this time on patient grew gradually weaker, and in spite of every effort to check the ravages of disease it steadily progressed toward a fatal termination. The discharge increased and became offensive.

May 14th.—My health having become impaired from overwork, aggravated by septicæmia (contracted from a wound upon the finger received during an operation), I was compelled to leave the city for recuperation. The case then passed into the hands of my friend, Dr. T. Eckles, who attended her until her death, which occurred in July, from exhaustion and the encroachment of the neoplasm upon the pelvic viscera. During all the subsequent course of the disease, no attempt was made to remove the growth, and the patient took but very little medicine and used but a small amount of food.

*Case VII.* I was called April 1st to see Mrs. K., in consultation with Drs. Seele and Lee. I found patient complaining of weight in pelvis, pain in back and loins, copious hæmorrhage. Examination showed the uterus enlarged to the size of pregnancy at the fifth month, and lifted above the superior strait. The os patulous and a copious sanio-purulent discharge constantly escaped. The finger, introduced into the os, encountered a semi-solid granular mass of peculiar feel. The sound entered with some difficulty, giving a sensation of contact with calcareous matter, and giving rise to copious hæmorrhage. Further examination convinced us that we had encountered the anomally of a "uterine stone," or calcified fibroid. The patient was then anæsthetized and attempts made to enucleate and remove the tumor. This signally failed, and I soon became convinced that the stone was too large to remove —*per vias naturales.* On consulting hastily with my colleague, laparo-elytrotomy was decided upon. This being my maiden effort in this direction, and, in fact, never having seen the operation, and only having cursorily read the report of Thomas' and Skeene's operations, I approached the case with some degree of awe. The hand was passed into the vagina and the relation of the parts learned. An incision was then made, extending from the crest of the right ilium to the border of the symphysis pubis. The epigastric artery was tied and cut and the peritoneum pushed up, and then an incision was made through the tissues to meet the finger in the vagina. The opening was now enlarged as much as possible and the os dilated still more and an attempt was made to grasp the stone and remove it. This, however, failed, from the size of the concretion. After incising the os and dilating the external incision as much as could be possibly done with safety and the impossibility of removing the stone in this manner ascertained, a silver retracter was bent so as to conform to the curve of the uterus, forced in between its walls and the calculus, and, with a fine finger saw, the mass was slowly and laboriously divided and removed in halves. Even this was so large that the tissues were lacerated in its removal. The uterus was then cleared of a mass of sarcomatous tissue, which surrounded the stone, and, in fact, which imperfectly merged from a true sarcoma into a granular calcareous mass. The uterus was next replaced, the walls of the incision brought together and secured with silver wire sutures coated with carbolized colodion and covered with a layer of carbolated cotton. The vagina was then washed out with carbolated water and a pledget of carbolated cotton placed therein and the patient put to bed and allowed to recover from the anæsthesia.

The patient endured the operation remarkably well. The shock was not excessive, the hæmorrhage, although profuse, was yet not exhausting. One grain of opium by the rectum was then given and five grains of quinine by the mouth. The patient rested well through the night and in the morning was remarkably comfortable.

I now left the patient in the care of Dr. Seele, the family physician, who reported as follows:

April 5th. — The patient doing finely; vaginal discharge copious and purulent with shreds of decomposing tissue. Reaction fully established. Temperature, 37.8°; pulse, 85; appetite good; urine, high colored and loaded with floculent matter. Continued iron and quinine.

April 9th. — The patient improving, but I find strong urinous smell in secretion from vagina. Rinsed out vagina and uterus with solution of carbolic acid and tannin.

April 14th. — Patient convalescing rapidly. Incision healed by first intention. Involution of uterus slow but satisfactory. The discharge gradually lessened and the urinous odor disappeared on the tenth or eleventh. The tumor proper, after removing the sarcomatous tissue surrounding it, measured 17½ Ctm. in its longer diameter and 14¼ Ctm. in its shorter. The weight was 4½ lbs. The tumor was composed of almost solid calculi of phosphate of lime and magnesia, carbonate of lime with interstitial fibers of fibrous tissue, albuminous shreds, etc. The surrounding magma consisted of sarcomatous tissue closely packed with cretaceous or calcareous accretions from the size of a pea to that of fine sand, being more closely deposited around the calculus and shading off towards the uterine parenchyma to a few scattering granules. There was estimated to be two or three pounds of this tissue.

## Figures and Tables

**Plate I, Case 1. f1:**
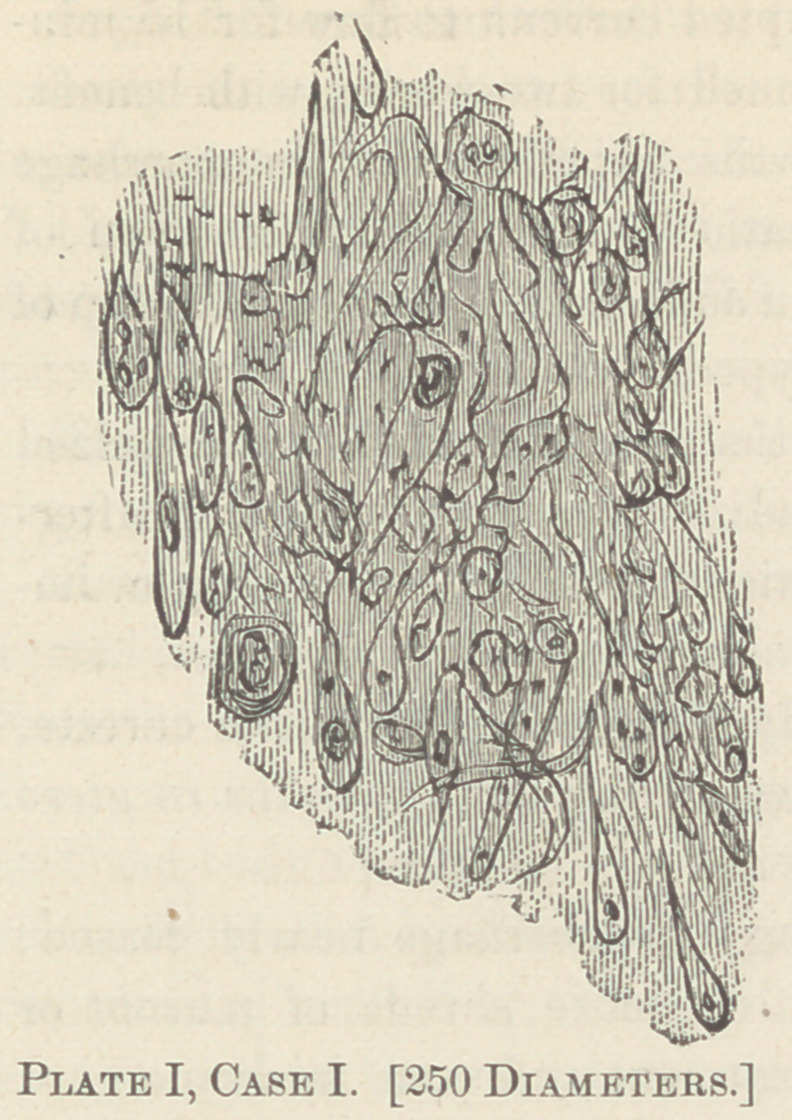


**Plate II, Case 4. f2:**
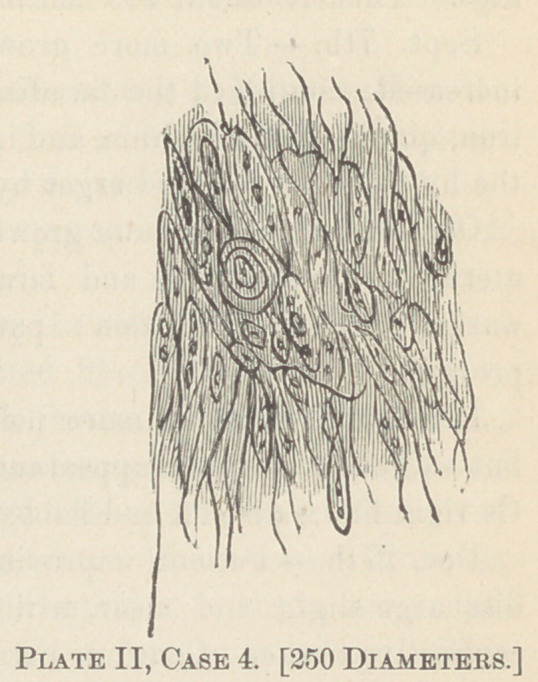


**Plate III, Case 3. f3:**
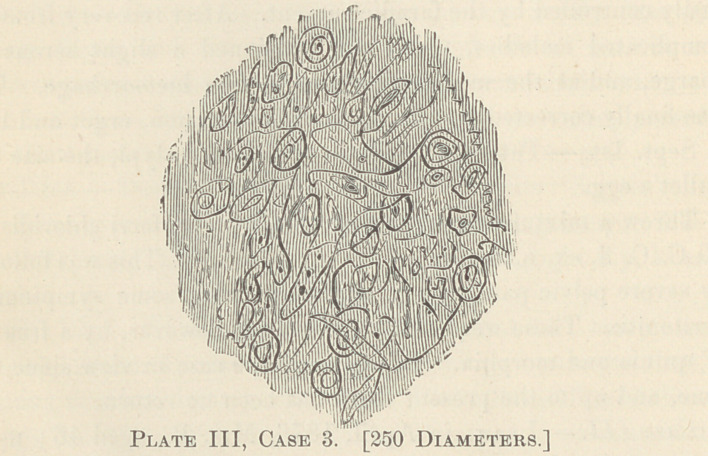


**Plate IV, Case 6. f4:**
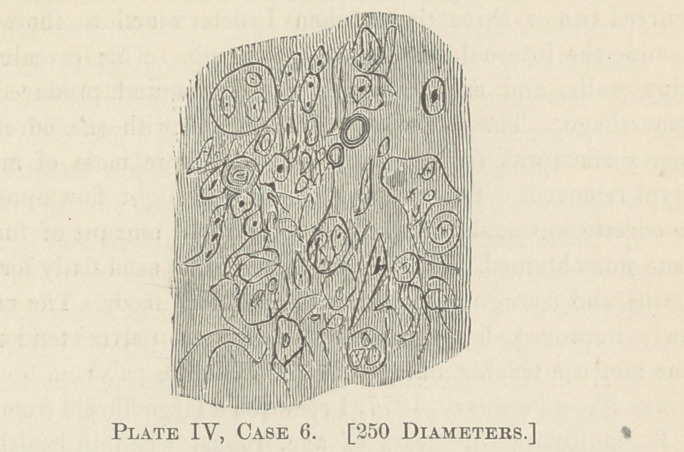


**Plate V, Case 5. f5:**